# Unusual Skin lesion

**Published:** 2012-12-10

**Authors:** Tom Reisler, Shahida Ahmed, Mirseyed Mohit-Tabatabi

**Affiliations:** ^a^Plastic and Reconstructive Surgery, Division of Department of General Surgery Veteran's Aff1irs, New Jersey Health Care System, South Orange; ^b^Pathology, Department of Veteran's Aff1irs, New Jersey Health Care System, South Orange; ^c^Surgical Oncology, Department of Veteran's Aff1irs, New Jersey Health Care System, South Orange

## DESCRIPTION

**Figure 1:**
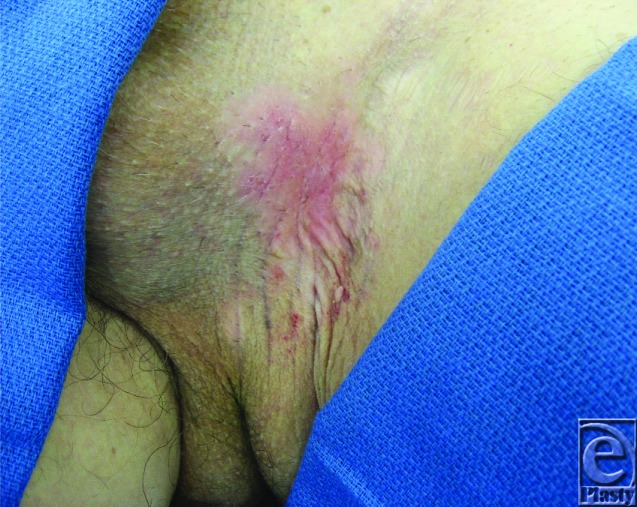
Exposure of the left groin skin lesion with the scrotum pulled to the right.

A 66-year-old white man presents with a 3-year history of a pruritic, burning, erythematous lesion in the left groin. It was treated in the past with topical antifungals without any improvement. Physical examination revealed a 7× 5-cm erythematous lesion in the left groin (Fig 1). A 3-mm punch biopsy was preformed.

## QUESTIONS

1. **What is the differential diagnosis?**
2. **What other underlying conditions may be present?**
3. **What are the treatment options?**

## DISCUSSION

**Figure 2:**
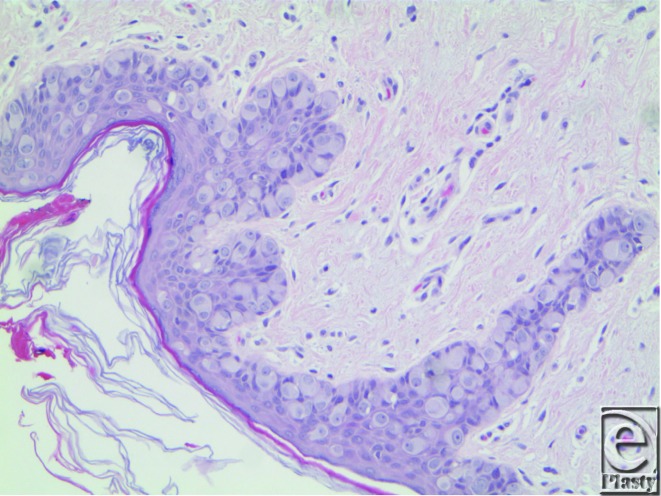
Large, pale, vacuolated single, and small nests pagetoid cells concentrated just above the basal layer of epidermis (H&E stain 60×).

The patient's skin lesion punch biopsy, and subsequent treatment with wide local excision, confirmed extramammary Paget's Disease (EMPD), which is a rare, slow-growing neoplastic condition (Fig 2) of apocrine gland-bearing skin that is thought to originate either from the intraepidermal cells of apocrine gland ducts or from pluripotent keratinocyte stem cells.^1^ Lesions of EMPD typically occur in sites with profuse apocrine glands, such as anogenital region, axilla, eyelids, scalp, and buttocks. Extramammary Paget's disease is usually noninvasive but sometimes shows aggressive behavior of local recurrence and coexisting internal malignancy. The location of the underlying internal malignancy appears to be closely related to the location of the EMPD. Extramammary Paget's disease of the perianal skin is often related to colorectal neoplasia and a penile-scrotal-groin location with genitourinary malignancy. In light of these associations, a thorough investigation for an underlying carcinoma should accompany every confirmed diagnosis of EMPD.^2^ The finding from our patient's endoscopic evaluation of the gastrointestinal and urinary tracts was negative, as well as no evidence of malignancy on abdominopelvic computed tomographic and positron emission tomography scans.

Extramammary Paget's disease generally presents between the ages of 50 and 80 years, and most commonly in white women. The most common presenting complaint is pruritus,^1^ but patients may experience burning, irritation, pain, tenderness, bleeding, and swelling.^2^ Clinically, lesions are well-demarcated, erythematous, or leucoplakic plaques with an eczematous appearance.^2^^,^^3^ Diagnosis is often missed or delayed, given the propensity of this lesion to be mistaken for fungal infection, contact dermatitis, lichen sclerosus, psoriasis, and Bowen's disease. The diagnosis is confirmed by biopsy.

Although surgical excision carries a high recurrence rate, it remains the standard therapy for EMPD.^4^ Local recurrence has been reported in around 40% to 50% of cases with equivalent rate of positive margin after surgical excision.^5^^,^^6^ However, it has also been reported in many cases with tumor-negative margin. Recurrence is associated with the tendency of EMPD lesions to have irregular margins and skipped tumor foci from either multifocal origin or intraepidermal metastasis. Localized treatment modalities such as conventional scalpel surgery, preexcisional mapping of the lesion with multiple punch biopsies, Mohs microsurgery, radiation, topical chemotherapy, photodynamic therapy, and carbon dioxide laser ablation may be appropriate for noninvasive, well-defined, unicentric EMPD.^3^ For patients with associated internal malignancy, surgery, in addition to chemotherapy and radiation, may be indicated.^4^^,^^7^ Our patient required a second wide local excision as the first wide local excision had one positive peripheral margin. All patients require a careful follow-up to detect recurrence or internal malignancy.
